# Adherence to diet with higher dietary diabetes risk reduction score is associated with reduced risk of type 2 diabetes incident in Iranian adults

**DOI:** 10.1186/s12889-023-16024-9

**Published:** 2023-06-14

**Authors:** Mitra Kazemi Jahromi, Hossein Farhadnejad, Farshad Teymoori, Golaleh Asghari, Mahsa Kalantari, Parvin Mirmiran, Fereidoun Azizi

**Affiliations:** 1 Endocrinology and Metabolism Research Center, Hormozgan University of Medical Sciences, Bandar Abbas, Iran; 2 Nutrition and Endocrine Research Center, Research Institute for Endocrine Sciences, Shahid Beheshti University of Medical Sciences, Tehran, Iran; 3 Department of Nutrition, School of Public Health, Iran University of Medical Sciences, Tehran, Iran; 4 Department of Clinical Nutrition and Dietetics, Faculty of Nutrition Sciences and Food Technology, National Nutrition and Food Technology Research Institute, Shahid Beheshti University of Medical Sciences, Tehran, Iran; 5 Endocrine Research Center, Research Institute for Endocrine Sciences, Shahid Beheshti University of Medical Sciences, Tehran, Iran

**Keywords:** Dietary diabetes risk reduction score, Diet quality, Type 2 diabetes, Iranian adults

## Abstract

**Background:**

The Dietary diabetes risk reduction score (DDRRS) has recently been considered by researchers as a diet quality index to predict the risk of chronic diseases, such as type 2 diabetes (T2D). In this study, we aimed to assess the association of DDRRS with T2D risk in Iranian adults.

**Methods:**

Subjects aged ≥ 40 years without T2D (n = 2081) were selected for the current study from participants of the Tehran Lipid and Glucose Study (2009–2011) and followed for a mean of 6.01 years. We used the food frequency questionnaire to determine the DDRRS that is characterized by eight components, including higher consumption of nuts, cereal fiber, coffee, and polyunsaturated to saturated fat ratio and lower consumption of red or processed meats, trans fats, sugar-sweetened beverages, and high glycemic index foods. The multivariable logistic regression analysis was used to determine the odds ratio (ORs) and 95% confidence interval (CI) of T2D across the DDRRS tertiles.

**Results:**

The mean ± SD age of individuals was 50.4 ± 8.2 years at baseline. The Median (25–75 interquartile range) DDRRS of the study population was 24(22–27). During the study follow-up, 233(11.2%) new cases of T2D were ascertained. In the age and sex-adjusted model, the odds of T2D were decreased across tertiles of DDRRS (OR = 0.68; 95%CI: 0.48–0.97, P for trend = 0.037). Based on the multivariable-adjusted model, after controlling all potential confounders, the risk of T2D is reduced across tertiles of DDRRS (OR = 0.66; 95%CI: 0.44–0.98, P for trend = 0.047). Also, higher scores (lower consumption) of red and processed meat (OR = 0.59; 95%CI: 0.39–0.88, P = 0.012) and sugar-sweetened beverages (OR = 0.49; 95%CI: 0.32–0.76, P = 0.002) as DDRRS components were associated with decreased T2D incident.

**Conclusions:**

Our findings suggested that a diet with a higher score of DDRRS may be related to reducing the risk of T2D in Iranian adults.

## Background

Type 2 diabetes (T2D) is a known major public health concern, with an adverse impact on life expectancy and health expenditures [1]. T2D as a complex metabolic disorder is recognized as one of the important preventable risk factors for the progression of cardiovascular disease (CVD) risk and mortality worldwide [2, 3]. Recent reports showed that in 2017 the global prevalence of T2D in the adult population was 8.5% (425 million people), and it has been forecast to reach 629 million by 2045 [2]. Also, based on national data from Iran, it was reported that the T2D incidence rate in adults was 36.3 per 1000 person-years, with more than 800,000 new cases per year [4]. Also, it is anticipated that in 2030, nearly 9.2 million Iranians will have T2D [5]. Insulin resistance and dysregulation of glucose metabolism as a consequence of impaired insulin action and secretion, along with metabolic susceptibility and genetic predisposition, have the main role in the pathogenesis of T2D [6]. Also, an inappropriate lifestyle, characterized by poor diet, smoking, physical inactivity, alcohol consumption, and elevated body mass index (BMI), is crucial in developing T2D [2, 3, 7].

Sufficient evidence revealed that lifestyle risk modification, such as improving dietary intakes, can be considered a strong strategy in the prevention of T2D [8], the findings of nutritional investigations suggested that the designing and implementation of dietary guidelines for the management of chronic diseases such as T2D based on evidence from studies that examined the role of a single food group or nutrient individually in the prediction of chronic diseases risk has limitations, whereas the results of studies conducted on the association of whole dietary patterns or diet quality scores with chronic diseases risk are more useful in this regard [9, 10]. Therefore, assessing the relationship between nutritional factors in the form of a single dietary pattern or diet quality score and the risk of chronic diseases may provide more useful evidence to achieve practical nutritional recommendations or interventions for the management of chronic diseases, such as T2D.

Several studies used various dietary indices such as the Mediterranean diet [11], low carbohydrate diet (LCD) [12], and dietary inflammatory index (DII) [13] to assess overall diet quality, which results of these studies have shown that a stronger adherence to the aforementioned dietary indices can be related to the risk of T2D. Also, in 2015, the Rhee et al. study [14] originated dietary diabetes risk reduction score (DDRRS) as a new diet quality index, which is characterized by high consumption of coffee, nuts, cereal fiber, the higher ratio of polyunsaturated: saturated fats (PUFA: SFA), and lower consumption of red and processed meats, dietary glycemic index (GI), sugar-sweetened beverages (SSBs) and trans fatty acids (TFA). To determine the total DDRRS score, for each dietary determinant of DDRRS, participants are categorized into quartiles based on their intake ranking, and component scores are considered for participants according to their ranks for each DDRRS component, which varies between 1 and 4 points. Finally, the DDRRS diet score is determined based on the sum of its eight dietary components score for individuals, which score range is 8 (poor diet) to 32 (healthy diet). Rhee et al. revealed that a higher score of DDRRS may decrease the risk of T2D [14]. Also, an inverse relationship was found between a higher score of DDRRS and the risk of metabolic syndrome [15] and cancer [16].

Considering that the association of DDRRS as a single set of different dietary factors and incident T2D in people of the Middle East and North Africa region, such as Iran, has not yet been studied; therefore, in the current study, we aimed to assess the association of DDRRS with incident T2D after 6.01 years of follow-up among Iranian adult.

## Methods and materials

### Research subjects and study design

This study was performed using data collected from the Tehran Lipid and Glucose Study (TLGS), a prospective population-based cohort study that was conducted on a representative urban population of Tehran, including 15,005 participants aged ≥ 3 years, to assess the risk of non-communicable diseases (NCD) and their related risk factors [17]. The first phase of TLGS was a cross-sectional survey initiated in March 1999. Data collection from participants, conducted prospectively at three years intervals, is ongoing; the details of the TLGS have been reported previously [17]. In the fourth survey of the TLGS (2009–2011), of 12,823 participants, 7956 subjects, aged 3–75 years, were randomly selected to be assessed for dietary intakes.

**Fig. 1:**
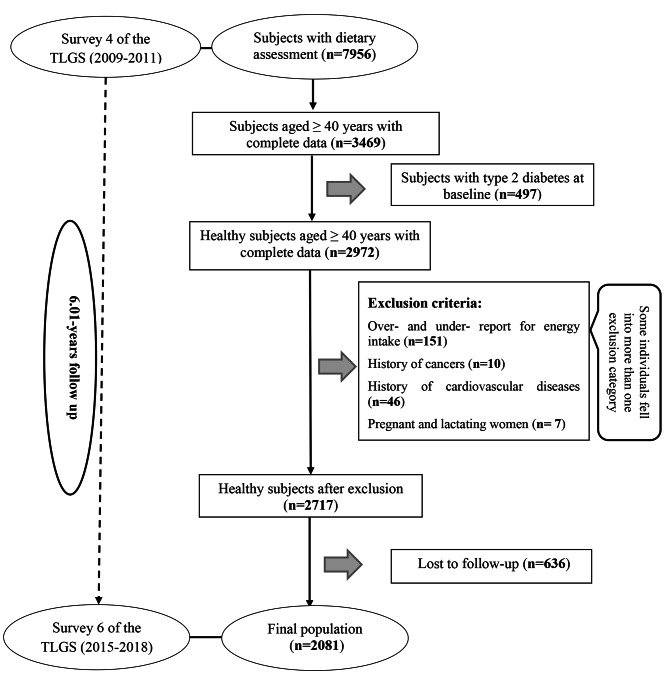
Flow chart of the Tehran Lipid and Glucose Study (TLGS) participants

For the current study, of 7956 subjects in the fourth survey of the TLGS (baseline examination), 3469 participants, aged ≥ 40 years, who had complete data on demographics, anthropometrics, clinical, biochemical, and dietary characteristics were enrolled. Subjects with under- or over-reported dietary intakes (< 800 kcal/day or > 4200 kcal/day, respectively) [18] or those were on specific diets (n = 151), those with missing data on diabetes diagnosis criteria specialty 2-hour blood sugar (n = 90), those with prevalent cancer (n = 10) and cardiovascular diseases (n = 46), and pregnant and lactating women (n = 7), were excluded; some of whom fell into more than one category. The participants with T2D at baseline (fourth survey) were also excluded (n = 497), then, 2717 healthy individuals (free of T2D) were followed until the sixth survey (2015–2018), for a mean period of 6.01 years from the baseline phase (fourth survey). Finally, after excluding the individuals who left the study (n = 636), final analyses were performed on the data of 2081 adults (Fig. 1).

The required sample size for the present study was calculated using the G Power software (3.1.9.4). Considering the confidence interval of 95% (α = 0.05) and study the power of 80%, diabetes incidence of 10%, and odds ratio of 0.6 for the highest vs. lowest tertiles of DDRS based on the results of previous studies among Iranian and US population which assessed the DDRS score relationship with metabolic syndrome [15] and diabetes [14], the required sample size calculated by 1872 participants. After applying the inclusion and exclusion criteria in the TLGS population in the fourth survey as the baseline survey, we reached 2081 subjects for final analysis.

Type 2 diabetes was defined according to the American Diabetes Association (ADA) criteria as fasting plasma glucose (FPG) ≥ 126 mg/dl or 2-h post-75-gram glucose load ≥ 200 mg/dl or using anti-diabetic medication [19].

### Dietary assessment

The dietary intake was determined using a valid and reliable 168-item semi-quantitative food frequency for the study population over the previous year at baseline [20]. The expert nutritionist asked individuals to report their consumption frequency for each food item during the past year on a daily, weekly, or monthly basis. We converted to grams the portion sizes of consumed foods reported in household measures. Considering that the Iranian Food Composition Table (FCT) is incomplete and has limited data on the nutrient content of raw foods and beverages, we used the United States Department of Agriculture (USDA) FCT [12], but for national foods not listed in the USDA FCT, the Iranian FCT [21] was used.

### DDRRS scoring

The score of DDRRS was calculated based on method scoring of the Rhee et al. study [14]; eight food components, including higher consumption of cereal fiber, nuts, coffee, and PUFA: SAFA ratio and lower consumption of red or processed meats, SSBs, TFA, and dietary GI were considered for computing DDRRS. For each dietary determinant, participants were categorized into quartiles based on their intake ranking, and component scores were considered for cereal fiber, coffee, nuts, and PUFA: SAFA ratio to determine the participant’s quartiles rankings, e.g., subjects in the lowest quartile received 1 point, and those in the highest quartile received 4 points. Scores were reversed for SSBs, red or processed meats, TFA, and dietary GI. Thus, participants in the lowest quartile were assigned a score of 4 points, and those in the highest quartile were assigned 1 point. Finally, the points for all eight items were summed up to calculate the DDRRS. The range score of total DDRRS was from 8 (poor diet) to 32 (healthy diet).

### Physical activity assessment

Data on physical activity were collected using a modifiable activity questionnaire (MAQ), which was previously modified and validated among Iranians [22]. We asked individuals to report the frequency and time spent on activities of light, moderate, hard, and very hard intensity during the past year, according to a list of common activities of daily life; physical activity levels of participants were reported as metabolic equivalent hours per week (MET-h/wk).

### Demographic and anthropometric measures

Trained interviewers used pretested questionnaires to collect participants’ data on demographic variables, medical history, drug use, and smoking habits at baseline (2009–2011). The body weight was measured to the nearest 100 g using digital scales, while participants were with minimal clothing and without shoes. Height was recorded to the nearest 0.5 cm using a tape meter, without shoes, and in a standing position. Body mass index (BMI) was computed as weight (kg) divided by the height in square (m^2^). We measured the waist circumference (WC) to the nearest 0.1 cm using a non-elastic tape measure, midway over light clothing, in the umbilical area, without any pressure on the body.

### Biochemical measures

Blood samples were taken from all participants after 12–14 h of overnight fasting in a sitting position based on the standard protocol. These samples were centrifuged within 30–45 min of collection. We performed all blood analyses at the TLGS research laboratory and analyzed samples using the Selectra 2 auto-analyzer (Vital Scientific, Spankeren, The Netherlands). FPG was assayed using glucose oxidase. We used an enzymatic colorimetric method to determine the level of FPG. Both inter-and intra-assay coefficient variations were 2.2% for FPG. The analyses were done using commercial kits (Pars Azmoon Inc., Tehran, Iran). In the TLGS, the 2-hour oral glucose tolerance test was performed using an 82.5-gram of glucose monohydrate solution (equivalent to 75 g anhydrous glucose), which was administered orally to individuals, except diabetic patients on anti-diabetic medication based on the prescription of the endocrinologist.

### Statistical analysis

All statistical analyses were performed using The Statistical Package for Social Sciences (Version 20.0; SPSS, Chicago, IL). The variables’ normality was checked using histogram charts and Kolmogorov–Smirnov test. We categorized participants according to tertiles of DDRRS cut-points; baseline characteristics of the individuals are expressed as the mean ± SD or median (25–75 interquartile) for quantitative variables and percentages for qualitative variables. We used linear regression and Chi-square to test the trends of continuous and categorical variables across DDRRS tertiles, respectively.

The multivariable logistic regression models were used to estimate the risk of 6.01 years incident T2D in each tertile of DDRRS, and the odds ratio (ORs) and 95% confidence intervals (CIs) were determined. The first tertile of DDRRS was considered as the reference group. Potential confounders were age, sex, family history of diabetes, physical activity, BMI, education level, occupation status, marital status, smoking, baseline FPG, and daily energy intake. To calculate the trend of OR across tertiles of DDRRS, we considered the tertile categories as continuous variables. P-values < 0.05 were considered to be statistically significant.

## Results

### General findings

The mean ± SD age and BMI of individuals were 50.4 ± 8.2 years and 28.3 ± 4.3 Kg/m^2^, respectively, and 53.4% of participants were women. The median (IQR) of DDRRS of participants was 24.0 (22.0–27.0). Also, median (IQR) scores of DDRRS components, including cereal fiber, PUFA/SFA ratio, nuts, red and processed meat, GI, SSBB, and TFA was 3.0 (2.0–4.0) in all participants. Furthermore, the median (IQR) coffee score was 3.0 (1.0–4.0). During 6.01 years of follow-up, 233 (11.2%) new cases of T2D were ascertained.

### Characteristics of participants according to tertiles of DDRRS

Table 1 shows the general baseline characteristics of the participants across tertiles of DDRRS. The mean HDL-C increased significantly across tertiles of DDRRS, whereas the % of smoking, % of employment, and mean of DBP and FPG decreased across tertiles of DDRRS (P < 0.05). No significant differences in age, physical activity, % of women, BMI, TGs, TC, LDL-C, SBP, and education level were observed in subjects according to tertiles of DDRRS.

**Table 1:** Baseline lifestyle and clinical characteristics of participants according to tertiles of dietary diabetes risk reduction score: Tehran Lipid and Glucose Study

Variables	Dietary diabetes risk reduction score tertiles			P _trend_
	Tertile 1 n = 1429	Tertile 2 n = 916	Tertile 3 n = 1216	
Age (years)	50.09 ± 8.14	50.44 ± 8.11	51.00 ± 8.36	0.060
Men (%)	47.4	47.7	44.5	0.433
Body mass index (kg/m^2^)	28.36 ± 4.35	28.25 ± 4.30	28.32 ± 4.38	0.861
Waist circumference (cm)	95.71 ± 10.68	95.21 ± 10.11	94.94 ± 10.25	0.182
Current smoker (%)	12.4	13.7	8.9	0.019
Academic education (%)	17.1	21.5	20.4	0.104
Employed (%)	86.8	83.4	81.8	0.040
Physical activity (MET h/week)	66.8 (32.7–99.8)	70.8 (35.7–99.5)	60.7 (32.7–92.2)	0.287
Fasting plasma glucose (mg /dl)	95.85 ± 8.78	95.25 ± 8.97	94.82 ± 8.79	0.036
2-hour plasma glucose (mg /dl)	108.95 ± 29.96	107.74 ± 28.65	106.78 ± 27.85	0.174
Family history of diabetes (%)	10.2	10.5	9.9	0.930

Data are represented as mean ± SD for continuous variables and percentage for categorical variables. Linear regression and Chi-square analyses were used for the test of trend continuous variables and categorical variables according to the tertiles of dietary diabetes risk reduction score, respectively

Also, Table 2 indicates the results on baseline dietary data of participants across tertiles of DDRRS. Dietary intakes of nuts, cereal fiber, coffee, polyunsaturated to saturated fat ratio, protein, carbohydrate, total dietary fiber, vitamin C, potassium, calcium, and magnesium significantly increased across tertiles of DDRRS (P for trend < 0.05), however, dietary intakes of red or processed meats, trans fats, SSBs, and high GI foods, total fats, SFA, and MUFA were decreased across tertiles of DDRRS score (P for trend < 0.05).

**Table 2:** Dietary intakes of the study population according to the tertiles of dietary diabetes risk reduction score

Variables	Dietary diabetes risk reduction score tertiles			P for trend
	Tertile 1 n = 660	Tertile 2 n = 782	Tertile 3 n = 638	
Dietary diabetes risk reduction score	20.0 (19.0–22.0)	24.0 (23.0–25.0)	28.0 (27.0–30.0)	
**DDRRS components**				
Cereal fiber (g)	6.10 (3.48–11.02)	8.03 (4.00-16.51)	10.31 (5.18–19.86)	0.001
Coffee (cup per week)	0.00 (0.00-0.02)	0.01 (0.00-0.18)	0.05 (0.00-0.27)	0.001
PUFA/SFA ratio	0.58 ± 0.22	0.65 ± 0.24	0.73 ± 0.23	< 0.001
Nuts (serving per day)	0.12 (0.05–0.26)	0.17 (0.07–0.34)	0.28 (0.12–0.48)	< 0.001
Red and processed meat (serving per day)	0.77 (0.48–1.23)	0.50 (0.32–0.78)	0.32 (0.21–0.50)	< 0.001
Glycemic index	216.3 ± 63.7	192.6 ± 69.8	169.0 ± 59.9	< 0.001
Sugar-sweetened beverages (serving per week)	2.05 (0.78-5.00)	0.97 (0.39–2.14)	0.49 (0.13–1.24)	< 0.001
Trans fatty acids (% energy)	3.66 (1.85–5.25)	2.00 (1.08–4.42)	1.15 (0.71–2.20)	< 0.001
**Other nutritional factors**				
Total energy intake (kcal/day)	2584 ± 647	2352 ± 709	2088 ± 619	< 0.001
Protein (% energy)	14.7 ± 5.2	15.0 ± 2.9	15.4 ± 2.5	0.002
Carbohydrate (% energy)	58.0 ± 6.6	59.0 ± 6.6	60.7 ± 6.7	< 0.001
Total fat (% energy)	30.7 ± 6.0	29.6 ± 6.5	28.0 ± 5.7	< 0.001
Saturated fat (% energy)	10.5 ± 2.8	9.5 ± 2.6	8.5 ± 2.3	< 0.001
Monounsaturated fat (% energy)	10.2 ± 2.5	9.9 ± 2.9	9.3 ± 3.1	< 0.001
Polyunsaturated fat (% energy)	5.8 ± 1.8	5.9 ± 2.0	6.0 ± 1.8	0.128
Dietary fiber (g/1000 kcal)	19.1 ± 6.6	20.8 ± 8.3	22.2 ± 6.7	< 0.001
Vitamin C (mg/1000 kcal)	74.3 ± 43.1	76.0 ± 39.2	84.2 ± 46.5	< 0.001
Potassium (mg/1000 kcal)	1896 ± 531	2028 ± 552	2157 ± 604	< 0.001
Calcium (mg/1000 kcal)	618.7 ± 203.3	645.3 ± 209.8	660.4 ± 220.7	< 0.001
Magnesium (mg/1000 kcal)	184.6 ± 37.1	203.5 ± 38.8	222.3 ± 39.2	< 0.001

Data are represented as mean ± SD for continuous variables and percentage for categorical variables. Linear regression was used for the test of trend continuous variables according to the tertiles of dietary diabetes risk reduction score

Polyunsaturated fatty acids (PUFA) to saturated fatty acids (SFA) ratio

### The OR of T2D in the participants according to tertiles of DDRRS

The OR of T2D in the study population across tertiles of DDRRS was reported in Table 3. Based on the age and sex-adjusted model, the odds of T2D were reduced across the tertiles of DDRRS (OR = 0.68; 95%CI: 0.48–0.97, P for trend = 0.037). Also, in the multivariable-adjusted model, after adjustment of all potential confounding factors, including age, gender, BMI, family history of diabetes, education level, dietary energy intake, occupation status, marital status, smoking, physical activity, and baseline FPG level, the 6.01-year incidence of T2D is decreased across tertiles of DDRRS score (OR = 0.66; 95% CI: 0.44–0.98, P for trend = 0.047).

**Table 3:** Odds ratio (95% CI) of type 2 diabetes across tertiles of dietary diabetes risk reduction score among Iranian adults

	Dietary diabetes risk reduction score tertiles			P _trend_
	Tertile 1 n = 660	Tertile 2 n = 782	Tertile 3 n = 638	
Median (IQR)	20.00 (19.00–22.00)	24.00 (23.00–25.00)	28.00 (27.00–30.00)	
Mean ± SD	19.86 ± 2.08	24.44 ± 1.09	29.03 ± 2.05	
Case/Total	84/660	90/782	59/638	
Model 1^†^	Ref	0.90 (0.64–1.23)	0.70 (0.49–0.98)	0.048
Model 2^‡^	Ref	0.88 (0.64–1.21)	0.68 (0.48–0.97)	0.037
Model 3 ^§^	Ref	0.85 (0.59–1.22)	0.66 (0.44–0.98)	0.047

†The crude model

‡ Adjusted for age and sex

§ Adjusted for model 2 and smoking status, total energy intake, body mass index, family history of diabetes, physical activity, education level, occupation status, marital status, and baseline fasting plasma glucose

Table 4 showed the ORs and 95% CIs of T2D according to tertiles of the DDRRS components scores among individuals. After adjustment of potential confounders including age, sex, smoking status, total energy intake, body mass index, physical activity, education level, occupation status, marital status, and baseline FPG, the odds of T2D for participants in the highest tertile of red and processed meat score (OR:59; 95%CI: 0.39–0.88, P:0.012) and SSBs score (OR:0.49; 95%CI: 0.32–0.76, P: 0.002) was lower than individuals in the lowest tertile of these DDRRS components score. However, the association of other DDRRS components score, including cereal fiber score (OR:0.80; 95%CI: 0.57–1.12, P: 0.872), coffee score (OR:0.79; 95%CI: 0.58–1.09, P: 0.389), PUFA/SFA ratio score (OR:1.00; 95%CI: 0.71–1.54, P: 0.714), nuts score (OR:0.73; 95%CI: 0.49–1.09, P: 0.122), glycemic index score (OR:0.96; 95%CI: 0.53–1.74, P: 0.920), trans fatty acids score (OR:0.81; 95%CI: 0.57–1.14, P: 0.236) with risk of T2D was not statistically significant.

**Table 4:** Odds ratio (95% CI) of type 2 diabetes across tertiles of dietary diabetes risk reduction components score among Iranian adults

	Dietary diabetes risk reduction components score tertiles			P _trend_†
	**Tertile 1**	**Tertile 2**	**Tertile 3**	
**Cereal fiber score**	Ref	0.87 (0.57–1.32)	0.80 (0.57–1.12)	0.872
**Coffee score**	Ref	0.82 (0.53–1.27)	0.79 (0.58–1.09)	0.389
**PUFA/SFA ratio score**	Ref	1.10 (0.80–1.52)	1.00 (0.71–1.54)	0.714
**Nuts score**	Ref	0.80 (0.54–1.19)	0.73 (0.49–1.09)	0.122
**Red and processed meat score**	Ref	0.75 (0.54–1.03)	0.59 (0.39–0.88)	0.012
**Glycemic index score**	Ref	1.08 (0.71–1.63)	0.96 (0.53–1.74)	0.920
**Sugar-sweetened beverages score**	Ref	0.74 (0.53–1.03)	0.49 (0.32–0.76)	0.002
**Trans fatty acids score**	Ref	0.87 (0.59–1.28)	0.81 (0.57–1.14)	0.236

†Adjusted for age, sex, smoking status, total energy intake, body mass index, physical activity, education level, occupation status, marital status, and baseline fasting plasma glucose

## Discussion

In the present study, the association of DDRRS with the risk of T2D has been assessed among Iranian adults in the framework of the TLGS study after a 6.01-year follow-up. Results suggested that a dietary pattern with a higher score of DDRRS may be related to reducing the risk of T2D. Also, we observed an inverse association between higher scores of some DDRRS components, including SSBs and red and processed meat, and the risk of T2D incident.

Recently, various epidemiological studies investigated the possible role of DDRRS in predicting chronic diseases risk, including cancers [23–25], chronic kidney disease [26], metabolic syndrome [15], cardiovascular disease [27], T2D [14], and mortality[28], that most of the results of these studies showed the beneficial effect of a diet with a high DDRRS score in reducing the incidence of the above-mentioned diseases. However, Asghari et al. did not observe a significant relationship between the high DDRRS and the risk of cardiovascular disease [27]. Our findings are consistent with the results of a cohort study conducted on the American population by Rhee et al. to assess the possible protective role of DDRRS against T2D risk [14]. Rhee et al. revealed that a greater score of DDRRS may be related to reduced risk of T2D in all racial and ethnic groups by more than 30% [14]. Three studies reported that a diet with greater score of DDRRS is associated with a reduced risk of cancers, such as pancreatic cancer and breast cancer in adult population [23–25]. Also, a in a large sample size population-based cohort study on US population, a diet with higher score of DDRRS may reduce the risk of all-cause mortality and cause-specific mortality (such as cardiovascular disease, and cancer) [28]. Furthermore, our results are in agreement with the findings of the other study in framework of TLGS that has reported that a diet with a high DDRRS score is related to reduced risk of metabolic syndrome independent of various confounding variables [15]. Therefore, according to the results of previous studies and the our study findings, a diet with high DDRRS score that focused on higher intakes of nuts, whole grain, coffee, and polyunsaturated fatty acids, and lower consumption of red and processed meat, SSBs, and high GI foods can significantly reduce the risk of T2D in adults. It should be noted that the evidence from our study is very valuable, because we conducted this study as the first study in the MENA region, which has a different food pattern and food culture than Western countries. In fact, our study as a study with exploratory findings in this area is the beginning of a new research path to conduct more studies on the possible relationship between this food index and the risk of cardio-metabolic disorders, such asT2D in the population of different communities and to obtain corroborating evidence.

The inverse association of DDRRS with T2D may be attributed partly to the cooperative role of its different components, including polyunsaturated fatty acids [29, 30], cereal fibers [31], coffee [32], and nuts [33] as positive components, and TFA [34], red and processed meat [35], simple sugar [36], and high GI foods [37] as negative components. Because according to convincing evidence, these above-mentioned positive or negative determinants of DDRRS can appear as a part of individuals’ dietary pattern as a predictor for the risk of T2D incident. Previous reports revealed that high intakes of fiber as an important component of DDRRS, along with low dietary GI, can reduce T2D risk by reducing fasting plasma glucose and insulin level, improving glycemic control, and reducing insulin resistance and obesity [31]. Mechanistic pathways explaining the role of high fiber intake in reducing the risk of type 2 diabetes include involvement in slowing down the absorption of nutrients, especially glucose, controlling appetite, energy homeostasis regulation, modulating systemic inflammation with a negative effect on the production of inflammatory markers, making beneficial changes in the gut microbiota, regulation of hormone secretion involved in nutrient metabolism, and subsequently improving glucose homeostasis [38–40]. Also, a diet with low GI can be related to reduced risk of T2D with possible intermediary mechanisms, including decreased body weight and reduced central obesity, and reduced glucotoxicity and lipotoxicity [37]. Furthermore, previous studies have suggested that the minerals and vitamins with antioxidant properties and other beneficial compounds found in coffee and nuts can justify the possible beneficial role of these food items in reducing the risk of T2D. Nuts and coffee are rich in vitamin E, vitamin B3, potassium, magnesium, fiber, unsaturated fatty acids, polyphenols, phytosterols, etc., which may have a protective role in T2D risk through mediating the inflammation and oxidative status, regulatory effect on adiponectin and leptin levels, improving endothelial function, reducing plasma triglycerides and insulin resistance [30, 32, 33, 41].

Lower consumption of red and processed meat, SSBs, and TFA as dietary compounds with adverse health effects in the form of a diet with a high DDRRS score can play a key role in preventing T2D. Red and processed meats rich in saturated fatty acids, heme-iron, and nitrites and nitrates, may increase the risk of metabolic disorders such as T2D through increasing weight gain, hyperinsulinemia, insulin resistance, dysregulation of plasma glucose concentration, and elevated inflammatory mediators and oxidative stress, increased nitrosamines production, [35, 42]. Also, higher TFA intake from an unhealthy diet may play an important role in the pathogenesis of T2D and metabolic syndrome by negatively impacting lipid profile levels, promoting systemic inflammation, weight gain, increased insulin-related disorders, inducing endothelial dysfunction, and increased visceral adiposity [34]. Furthermore, higher intakes of SSBBs may individually play an important role in the prediction of T2D risk due to high added sugar content, incomplete compensatory reduction in intake of energy, weak effect on satiety, which create a positive energy balance status, and increased weight gain [43]. In general, the positive and negative determinants of DDRRS indicate that a diet with a high DDRRS score is rich in antioxidant nutrients, fiber, and phytochemical that mediates the effects of this dietary index on T2D risk by possible mechanisms, such as antioxidant, anti-inflammatory, and anti-atherogenic features, reducing visceral adiposity, and beneficial effects on glycemic control and insulin secretion and its function.

Several strengths of our study deserve to be mentioned. To the best of our knowledge, the current study is the first study with a prospective design to assess the possible link of DDRRS with the risk of T2D with a long-term follow-up in the MENA region. Also, we used valid and reliable food-frequency and physical activity questionnaires to collect the participant data on dietary intake and physical activity level. However, the present study has some limitations; although, like in epidemiological studies, in this study, we used validated questionnaires for dietary and physical activity assessment, so some measurement errors are expected.

The consumption of coffee and nuts as components of DDRRS in our population is low, which could limited our study to identify precisely a diet with a high DDRRS score in the study population. Also, despite controlling various major confounding variables in our analyses, there may still be residual or unmeasured confounders, the effects of which cannot be ruled out.

## Conclusions

In conclusion, our findings revealed that a dietary pattern with a higher DDRRS score, characterized by higher intakes of nuts, whole grain, coffee, and polyunsaturated fats, and a lower intake of red and processed meat, SSBs, and high GI foods, can be associated with reduced T2D risk.

## Data Availability

The datasets analyzed in the current study are available from the corresponding author on reasonable request.

## List of abbreviations

BMI: Body mass index
CI: confidence interval
CVD: cardiovascular disease
DBP: diastolic blood pressure
DDRRS: dietary diabetes risk reduction score
FCT: Food Composition Table
FFQ: food frequency questionnaire
FPG: Fasting plasma glucose
GI: glycemic index
HDL-C: high-density lipoprotein cholesterol
MAQ: modifiable activity questionnaire
MET-h/wk: metabolic equivalent hours per week
OR: odds ratio
TFA: Trans fatty acids
T2D: type 2 diabetes
P: S:polyunsaturated to saturated fats
PUFA: polyunsaturated fatty acids
SFA: saturated fatty acids
SSBs: sugar-sweetened beverages
TC: Total cholesterol
TGs: Triglyceride
TLGS: Tehran Lipid and Glucose Study
SBP: systolic blood pressure
SPSS: Statistical Package for Social Sciences program
